# Antimicrobial Resistance of Coagulase-Positive *Staphylococcus* Isolates Recovered in a Veterinary University Hospital

**DOI:** 10.3390/antibiotics9110752

**Published:** 2020-10-29

**Authors:** Marta Pérez-Sancho, Sergio Alvarez-Perez, Teresa Garcia-Seco, Marta Hernandez, David Rodríguez-Lázaro, Lucas Domínguez, Marta Eulalia García, Jose Luis Blanco

**Affiliations:** 1 Department of Animal Health, Faculty of Veterinary Medicine, Complutense University of Madrid, 28040 Madrid, Spain; sergioaperez@ucm.es (S.A.-P.); lucasdo@ucm.es (L.D.); megarcia@vet.ucm.es (M.E.G.); jlblanco@ucm.es (J.L.B.); 2 VISAVET Health Surveillance Centre. Complutense University, 28040 Madrid, Spain; teresagsr@visavet.ucm.es; 3 Veterinary Teaching Hospital, Faculty of Veterinary Medicine, Complutense University, 28040 Madrid, Spain; 4 Laboratorio de Biología Molecular y Microbiología, Instituto Tecnológico Agrario de Castilla y León, 47071 Valladolid, Spain; ita-HerPerMa@itacyl.es; 5 Área de Microbiología, Departamento de Biotecnología y Ciencia de los Alimentos, Universidad de Burgos, 09001 Burgos, Spain; drlazaro@ubu.es

**Keywords:** SIG group, MALDI-TOF, whole genome sequencing, antimicrobial resistance

## Abstract

The *Staphylococcus pseudintermedius* group (SIG) is an emerging threat in veterinary medicine, particularly methicillin-resistant (MRSP) isolates, which are frequently associated with multidrug resistance. Reliable identification of SIG members is critical to establish correct antimicrobial treatments. However, information on the molecular epidemiology and antimicrobial resistance patterns of MRSP in some regions is still limited. This study aimed to assess the antimicrobial resistance of SIG isolates recovered from animals at the Veterinary Teaching Hospital of Complutense University of Madrid (Spain) during a 10-year period (2007–2016). A total of 139 selected *Staphylococcus* isolates were subjected to species-level identification by different bioanalytical techniques (PCR, VITEK, MALDI-TOF) and subsequent antimicrobial susceptibility testing. Methicillin-resistant isolates (*n* = 20) were subjected to whole genome sequencing for further characterization of their antibiotic resistance determinants. Our results showed that there was a good correlation between PCR and MALDI-TOF identification, whereas VITEK showed very divergent results, thus confirming MALDI-TOF as a good alternative for species-level identification of coagulase-positive staphylococci. Notably, *S. pseudintermedius*, including the epidemic MRSP genotype ST71, was the only SIG species found among canine isolates. In addition, we found a high prevalence of multidrug resistance and resistance to fluoroquinolones, cephalosporins and macrolides. Finally, diverse genes associated with antibiotic resistance were detected among MRSP isolates, although the genetic basis of some of the resistant phenotypes (particularly to fluoroquinolones) could not be determined. In conclusion, our study reveals the circulation of MRSP in the veterinary setting in Spain, thus highlighting the emerging threat posed by this bacterial group and the need for further epidemiological surveillance.

## 1. Introduction

*Staphylococcus aureus* is a well-recognized pathogen that is responsible for a plethora of clinical manifestations (from skin infections to endocarditis, including fatal outcomes) in humans and animals worldwide. Coagulase-positive *Staphylococcus* (CoPS) isolates other than *S. aureus*, including the members of the *Staphylococcus intermedius* group (SIG, namely, *S. intermedius*, *S. pseudintermedius* and *S. delphini*) are emerging as a serious threat in veterinary medicine, especially in small animals. In particular, *S. pseudintermedius* is now considered the main CoPS species recovered from household pets [1]. *S. pseudintermedius* is also part of the normal microbiome of small animals and is considered an opportunistic pathogen that is implicated in a variety of infections (skin, uterus, urinary tract, abscesses, etc.) [2]. Additionally, *S. pseudintermedius* may pose a serious but often neglected zoonotic risk [3]. 

Methicillin-resistant *S. aureus* (MRSA) strains have become a major cause of concern in animal and human medicine. Similarly, decreased antimicrobial susceptibility has been detected in *S. pseudintermedius* isolates from companion animals. Strains carrying methicillin resistance genes (methicillin-resistant *Staphylococcus pseudintermedius*, MRSP) are of special importance in animal and human pathology [1]. Detailed molecular characterization of dominant MRSP strains may increase our knowledge about their distribution, epidemiology, pathogenesis and potential transmission in a given region. For example, certain multi-locus sequence typing (MLST) clonal complexes of MRSP have characteristic antimicrobial resistance profiles [4]. MRSP often display multidrug resistance to different antibiotics used in veterinary applications [1]. This may pose a challenge for veterinarians due to the scarcity of alternative antibiotics that are suitable for the treatment of MSRP infections [4]. In addition, knowledge about the carriage of antimicrobial resistance marker genes by MRSP isolates provides insights into the genetic basis of phenotypic resistance and may lead to the optimal selection of antibiotics for therapy. 

Correct species-level identification of the staphylococci is a critical step for the establishment of adequate antimicrobial treatments, and is key for successful clinical outcomes. In this sense, oxacillin minimum inhibitory concentration (MIC) breakpoints depend on the *Staphylococcus* species [5], which emphasizes the relevance of reliable species identification. In particular, the correct discrimination of *S. intermedius* and *S. pseudintermedius* is essential to clarify important aspects of their epidemiology [3]. Currently, there are no commercial rapid systems available for CoPS differentiation, which hinders their correct species-level identification in most diagnostic laboratories. In addition, different CoPS species cannot be distinguished by unique biochemical traits. Although molecular-based identification of CoPS isolates has been proposed using different molecular targets [6,7], including these methods in routine laboratory testing is highly demanding with regard to time and cost [5].

In recent years, the clinical relevance, molecular epidemiology and antimicrobial resistance patterns of MRSP have been assessed in different countries [8,9]. However, to our knowledge, no systematic study dealing with these issues has been carried out in Spain in domestic animals, and the scarce data currently available refers to healthy dogs [10,11,12]. Thus, the present study aimed to assess the prevalence and characteristics of *S. pseudintermedius* isolates recovered from animals at the Veterinary Teaching Hospital of Complutense University in Madrid (Madrid, Spain) over a period of ten years (2007–2016). Isolates were identified to the species level, and phenotypically and genotypically characterized. 

## 2. Results

### 2.1. Origin and Identification of Coagulase-Positive Staphylococcus Isolates

Most Staphylococcus isolates analyzed in this study were recovered from dogs (128/139, 92.1% of the total), but isolates also came from other sources including horses (n = 4, 2.9%), cats and mice (n = 2, 1.4% each), and lion, sheep and swine (n = 1, 0.7% each). Isolates were collected from ear exudates (n = 58, 41.7%), body fluids (urine, exudates from different origins, n = 38, 27.3%) or other origins (nails, hair, abscesses and other samples, n = 43, 31%). The results of the different CoPS identification techniques used in this study are summarized in Table 1. There was a good agreement for *S. pseudintermedius* (n = 117) identification by PCR and MALDI-TOF (κ = 0.528). In contrast, concordance between PCR and VITEK was much lower (κ = 0.010). Considering MALDI-TOF as the gold standard technique for *S. aureus* identification, VITEK identified correctly all isolates but one, which was assigned to *S. intermedius*. However, VITEK misidentified 13 *S. pseudintermedius* and one *S. schleiferi* isolates as *S. aureus*. All three *S. schleferi* isolates were identified by PCR and MALDI-TOF. In general, the correlation between PCR and MALDI-TOF results was good while VITEK showed very divergent results.

### 2.2. Antimicrobial Resistance Profiles

The results of the antimicrobial susceptibility test are shown in Table 2. Almost all CoPS isolates (131/139, 94.24%) were resistant to at least one of the antimicrobials tested. Notably, 50 different antimicrobial resistance profiles were detected. While 33 CoP isolates (24 *S. pseudintermedius* and 9 *S. aureus*) showed resistance exclusively to penicillin, the most frequent combination of antimicrobial resistance was tetracycline/penicillin (n = 19, all *S. pseudintermedius*), and 66 isolates (47.48%, 60 *S. pseudintermedius* and 6 *S. aureus*) were multidrug resistant (MDR), i.e. resistant to at least three distinct classes of antimicrobial agents. The trend in the proportion of AMR-CoPS isolates over time was assessed by comparing the number of resistant isolates at three different periods covering a similar number of isolates: 2007-2010 (n = 44), 2011-2014 (n = 44) and 2015-2016 (n = 51). Among all of the tested antimicrobial agents, ciprofloxacin (*P* = 0.013), norfloxacin (*P* = 0.02), ofloxacin (*P* = 0.013) and trimethoprim-sulfamethoxazole (*P* = 0.029) showed increasing resistance rates over time (Cochran–Armitage Test) (Table 3). 

The association between AMR and the clinical origin of the isolates (fluids, ear exudates or other sources) was not significant (*P* > 0.05, multiple proportions Z-test), except for gentamicin (*P* ˂ 0.05), for which 27.6% isolates from ear exudates but only 10.5% of isolates from body fluids were resistant to this antibiotic.

The three *S. schleiferi* isolates were susceptible to all antimicrobials except one isolate (strain 21S), which was resistant to fluoroquinolones (ciprofloxacin, ofloxacin and norfloxacin). *S. pseudintermedius* showed significantly higher resistance rates to kanamycin (*P* = 0.006), tetracycline (*P* = 0.001), trimethoprim-sulfamethoxazole (*P* = 0.003), clindamycin (*P* = 0.04) and chloramphenicol (*P* = 0.013) than *S. aureus*. However, *S. aureus* isolates were significantly more resistant to amikacin (*P* = 0.009) than *S. pseudintermedius*.

### 2.3. Genomic Characterization of the Methicillin-Resistant Coagulase-Positive Staphylococcus (MRCoPS)

A total of 20 CoPS isolates resistant to oxacillin and with a positive result in the ALERE PBP2a test (17 *S. pseudintermedius* and 3 *S. aureus* isolates) were subjected to molecular analysis. All the strains were isolated from dogs, except S. aureus strain 94S, which was isolated from a pig’s lung. There was a significant association between MDR and methicillin resistance for *S. pseudintermedius* isolates (*P* ˂ 0.05, Fisher’s exact test). MRSP and MSSP isolates showed resistance against the same antibiotics except in the case of amikacin, which was only detected in one MRSP isolate. Eighteen isolates (90%) were found to possess the methicillin resistance-encoding *mec*A gene (this gene was not found in the genomes of *S. pseudointermedius* isolates 125S and 134S) (Supplementary Material). We performed an in silico Staphylococcal cassette chromosome mec (SCC*mec*) analysis of the 18 *mec*A-containing isolates and found that most *S. pseudointermedius* isolates belonged to the SCC*mec* III type (3A) (13 isolates, 86.7%), whereas two isolates belonged to the SCC*mec* V type (13.3%). Likewise, three different sequence types (STs) were detected among MRSP isolates: ST71 (15 isolates, 88.23%), ST196 (1 isolate, 5.88%) and ST261 (1 isolate, 5.88%). Interestingly, there was a perfect correlation between SCC*mec* types and STs, as all SCC*mec* III (3A) isolates belonged to ST71, whereas MRSP isolates of the SCC*mec* V (2B) and SCC*mec* V types belonged to STs 261 and 196, respectively. Regarding the MLST results of MRSA isolates, one of the isolates (94S) belonged to ST398, which is the ST typically associated with the livestock-associated MRSA (LA-MRSA) lineage, whereas the other two MRSA isolates belonged to two other different lineages, namely, community-associated MRSA (CA-MRSA) lineage (isolate 55S, ST8), and hospital-associated MRSA (HA-MRSA) lineage (16S, ST125). 

The molecular AMR profiles of 20 MRCoPS are summarized in Table S1 (see Supplementary Material). A total of 17 out of 20 (85%) MRCoPS isolates carried the β-lactamase gene *blaZ*. The presence of genes encoding resistance to macrolides (clarithromycin and erythromycin) were detected in 18 MRCoPS isolates (90%) including 15 (75%) methylase gene *erm(B)*, and 2 (10%) methylase gene *erm(C)*. In addition, the ABC transporter-encoding gene *msrA* and modifying enzyme *mphC* gene were found in the same MRSA isolate, while *erm(B)* or *erm(C)* genes were not detected. Macrolide resistance-encoding genes were not detected in two MRSP. Moreover, detection of lincosamide nucleotidyl transferase-encoding gene, *lnu(A)* and *linA* genes were detected in only one MRSA isolate. Membrane-associated efflux pump genes *tet(K)* and ribosome binding site gene *tet(M)* were detected in five (25%) and three (15%) MRCoPS isolates. One MRSA isolate simultaneously carried *tet(K)* and *tet(M)* genes.

Results for aminoglycoside-streptothricin resistance-encoding genes are summarized in Table S1. Almost all MRCoPS isolates (17, 85%) carried the kanamycin resistance gene *aph(3´)*. In at least 90% of isolates, two aminoglycoside-streptothricin resistance-encoding genes were simultaneously detected with *ant(6) + aph(3’) + aad(6) + SAT-4* being the most frequent combination (15 out of 20) among MRCoPS in our study. The *cat(pc221)* chloramphenicol acetyltransferase gene was detected in the four chloramphenicol-resistant MRSP isolates. Finally, 80% of the isolates (16 MRSP) carried the trimethoprim resistance gene *dfr(G)*.

## 3. Discussion

*Staphylococcus pseudintermedius* has been identified as the most important SIG pathogen in dogs [1], although it may also be isolated from other animal species [13] and humans [14]. Furthermore, MRSP is frequently recovered from pets with pyoderma and healthy animals [9]. In this context, reliable and accurate species-level identification of *S. pseudintermedius* is key to the implementation of control measures for successful treatment and disease management [15]. Additionally, correct identification of *S. pseudintermedius* may help to clarify the current clinical role of this species in veterinary medicine [16].

In this study, *S. pseudintermedius* was the only SIG species found among canine isolates, which agrees with the results of previous studies [17]. Different authors have demonstrated that *S. pseudintermedius* is frequently misidentified as *S. intermedius* by phenotypic methods [5]. Indeed, 13 *S. pseudintermedius* isolates from our strain collection were misidentified as *S. aureus* and 85 as *S. intermedius* by the VITEK system. A possible explanation for these discrepancies could be the lack of β-galactosidase test in the VITEK panel, which is frequently used to differentiate between *S. pseudintermedius* and *S. aureus* [18]. Similarly, *S. pseudintermedius* isolates can be misidentified as *S. intermedius* based on mannitol fermentation tests [19]. Previous reports have recognized the *nuc*-based multiplex PCR assay described by Sasaki et al [5] as the gold standard method to differentiate CoPS species [20]. The suitability of MALDI-TOF approaches for species-level differentiation of SIG members has also been suggested [21], although certain limitations in identifying *S. pseudintermedius* were reported [20]. Accordingly, four out of 117 *S. pseudintermediu*s isolates included in this study were misidentified as *S. intermedius* by PCR. 

Regarding the genetic diversity of the MRSP isolates characterized in this study, it is notable that most of them (15 out of 17 isolates, i.e., 88.2%) belonged to the same sequence type (ST71), which is perfectly correlated with the SECCmec III(3A) type in those isolates containing the *mec*A gene. This contrasts with the results of previous studies that found a higher genetic diversity for *S. pseudintermedius* (e.g., 24 different STs out of 69 MRSP isolates and 11 STs out of 23 MRSP isolates [9,22]. The high prevalence of the ST71 genotype among the *S. pseudintermedius* isolates recovered from dogs in Spain has been previously reported [10]. Moreover, in some cases, it has been demonstrated that MRSP are often genetically more homogeneous than MSSP [23]. The ST71 clonal group has been formerly considered as epidemic in Europe, but this multidrug resistant lineage has also spread to other regions [24]. With respect to the two other MRSP genotypes detected in this study, ST196 has been found in Portugal [8]. On the other hand, molecular characterization revealed that our MRSA isolates belonged to three different STs (one isolate each), namely, ST398, ST128 and ST8, which represent two different origins: animal (ST398 belongs to the LA-MRSA linage), and human (ST8 and ST125 belong to CA-MRSA and HA-MRSA lineage, respectively). These findings on MRSA suggest that while isolate 94S, isolated from a pig’s lung, is a natural animal MRSA strain, the other two MRSA isolates isolated from dogs might have been transferred from the animals’ owners.

*S. pseudintermedius* isolates had the greatest levels of resistance to penicillin (104/117 isolates, i.e., 88.8%). The resistance rates to fluoroquinolones detected in the present study (around 20%) are in accordance with previous reports [25]. 

The methicillin resistance of our selected isolates was confirmed by a PBP2a-based culture colony test, which has been recognized as a suitable diagnostic test to detect beta-lactam-resistant SIG isolates [26]. Methicillin-resistant *S. aureus* and *S. pseudintermedius* have emerged in the last decade. The relevance of MRSP in veterinary clinics is related to their frequent multidrug resistance, even to all licensed drugs available for dogs [1]. In addition, MRSP is considered a nosocomial pathogen in veterinary care settings [27]. In particular, around 16% of the CoPS isolates recovered from veterinary patients and characterized in the present study were methicillin-resistant, thus confirming MRSP circulation among Spanish veterinary patients. The prevalence of MRSP found in our study is higher than the figures previously reported in Spain (1% in healthy household-dogs and 8% in healthy pound-dogs [10]). In contrast, high rates of MRSP isolation from veterinary patients have been reported in other countries [28].

Different studies have highlighted the emergence of multidrug-resistant CoPS [29], which were detected in 47.48% of isolates in the present study. Of these, 19 isolates were MRSP (out of 19, that is, 100%). MRSP are commonly associated with MDR profiles. For example, Gomez-Sanz et al. [10] found that all nine MRSP identified among 196 CoPS tested isolates were MDR. Different studies have also detected MDR among MSSP isolates [8]. MSSP isolates carried similar antibiotic resistance profiles to those previously reported in Spain showing high rates of resistances against *β*-lactams, macrolides, lincosamide and aminoglycosides [10,11]. In our study, 41.84% of MSSP isolates showed a multidrug-resistant profile, which is similar to 39% of MSSP isolates previously reported in Spain [11]. MSSP isolates are usually resistant to penicillin/ampicillin and tetracycline [30,31]. However, in our study, MSSP showed resistance to the same antimicrobials as MRSP except amikacin. This latter result agrees with that reported by Gold et al. [32], who found that resistance to amikacin was linked to methicillin-resistance.

Most antibiotic resistance genes detected in the genome of our collection of MRSP isolates have been reported by other authors and are presumed to be acquired via transposon transfer from other Gram-positive bacteria [33]. However, the genetic basis of some of the resistant phenotypes found in this study could not be determined. Previous studies have revealed discrepancies between antibiotic resistant phenotypes and those inferred from genetic information, particularly among isolates from some clonal complexes such as CC71 [34]. The lack of resistance breakpoints for veterinary isolates of *S. pseudintermedius* may contribute to these discrepancies [34].

The presence of the *mecA* gene in the genome of *S. pseudintermedius* isolates has been previously reported [29]. In our study, all but two (15/17, i.e., 88.2%) methicillin-resistant CoPS isolates had such antibiotic resistance markers. With regard to genes linked to tetracycline resistance, *tet(K)* gene was the most frequent gene in the set of MRSP isolates included in this study, which agrees with the results obtained by other authors (e.g., Perreten et al. [1]). It has been suggested that MRSP ST71 only showed *tet(K)* [8], which would explain its presence in most of our isolates. Actually, *tet(M)* was only present in the ST196 and ST261 isolates (one isolate each) that we found, and *tet(L)*, previously found in MSSP isolates from Spanish dogs [12], was not detected in any of the isolates characterized in this study.

As in other studies, the *cat(PC221)*-carrying plasmid was the most frequent mechanism of chloramphenicol resistance identified in our collection of *S. pseudintermedius* isolates [1]. As previously observed in Europe and North America, most of our MRSP and MRSA isolates carried the *blaZ* gene. Furthermore, the *aph(3’)* gene, which encodes an amynoglycoside-modyfying enzyme was the most frequent aminoglycoside resistance encoding gene found in isolates from dogs, thus agreeing with previous reports [32]. Remarkably, the streptomycin adenyltransferase encoding gene *ant(6)*, the kanamycin resistance-encoding gene *aph(3’)*, and the streptothricin acetyltransferase encoding gene *sat-4* were simultaneously detected in 65% of our CoPS isolates, which is a lower frequency than in previous reports from Spain [10] and other countries [1]. Previous studies have also detected the rRNA methylase-encoding gene *erm(B)* as the most frequent marker of macrolide/lincosamide resistance [35]. The dihydrofolate reductase-encoding gene *dfr(G)* was detected in most trimethoprim-resistant MSRA and MRSP isolates as previously found [10,13]. 

There is a notably high degree of heterogeneity in antibiotic resistance results across different studies focused on *S. pseudintermedius*, which may be due to the lack of harmonized strategies for antimicrobial susceptibility testing and the interpretation of resistance, as previously suggested by Moodley et al. [30]. Our study highlights the emergence of antibiotic resistance, particularly to fluoroquinolones and trimethoprim-sulfamethoxazole, for which an increasing resistance over time was demonstrated in CoPS isolates recovered from animals in Spain. The establishment of harmonized tools for CoPS surveillance may help to monitor the emergence of AMR in different countries, which is a key step for implementing adequate antibiotic management practices in veterinary clinics.

## 4. Materials and Methods

### 4.1. Samples and Isolates

A total of 5468 clinical samples were subjected to bacteriological analysis at the Veterinary Teaching Hospital of Complutense University of Madrid (Madrid, Spain) from 2007 to 2016. *Staphylococcu*s spp. was identified in 842 samples (15.4%) according to VITEK results (BioMèrieux, Marcy-l’Etoile, France). Of these, 139 isolates (16.5% of the total number of *Staphylococcus* isolates) were randomly selected for the present study for species-level identification by different bioanalytical techniques and subsequent antimicrobial susceptibility testing (as detailed below). 

### 4.2. Bacterial Identification

After VITEK identification, all studied isolates were subjected to MALDI-TOF mass spectrometry analysis, following the procedures detailed in Perez-Sancho et al. [36]. Briefly, isolates were grown on Columbia blood agar plates (bioMérieux) at 37 °C for 24 h. Proteins were extracted following the manufacturer’s instructions (Bruker Daltonik, Bremen, Germany). Spectra acquisition was performed automatically using an UltrafleXtreme device (Bruker Daltonik) in the linear and positive mode. Species-level identification was achieved by comparing the MALDI profile of isolates with those included in the BDAL Bruker database (5989 entries, accessed on April 2016) using MALDI Biotyper software (Bruker Daltonik). 

Additionally, SIG isolates were also identified by a multiplex PCR based on thermonuclease-encoding (*nuc*) gene [5], which is considered to be the gold standard technique for SIG species-level identification [20,37]. On the contrary, *S. aureus* isolates were only identified by MALDI-TOF MS, as this technique achieves reliable identification of this *Staphylococcus* species [38,39]. 

### 4.3. Antimicrobial Susceptibility Testing

Antibiotic susceptibility was assessed by the disk diffusion method following the Clinical and Laboratory Standard Institute (CLSI) guidelines [40,41,42]. MICs to vancomycin and oxacillin were determined by the agar dilution method, which was performed according to CLSI’s instructions [41]. Oxacillin-resistant isolates were subjected to the ALERE PBP2a culture colony test (Alere Healthcare, L’Hospitalet de Llobregat, Barcelona), which is an immunochromatographic membrane assay that allows the detection of the penicillin-binding protein PBP2a in clinical isolates in approximately 5 min. *Staphylococcus aureus* ATCC 25923 was included as the quality control strain in all these analyses.

### 4.4. Molecular Analysis of Methicillin Resistant CoPS

All isolates resistant to oxacillin and with a positive result in the ALERE PBP2a test were subjected to whole genome sequencing (WGS) for further characterization of their antibiotic resistance determinants. Bacterial strains were sequenced on a MiSeq (Illumina) platform as previously described [43]. Briefly, genomic DNA was purified from pure cultures with the QIAGEN DNeasy Blood and Tissue Kit, and sequencing libraries were prepared using the Nextera XT kit and sequenced using v3 reagents with 2 × 300 cycles. Raw reads were analyzed using the in-house bioinformatics pipeline TORMES® [44]. Briefly, it consists of quality filtering by Prinseq v.0.20.4 [45] and Trimmomatic [46]. Genomes were assembled by using SPAdes v3.10 [47], and classified taxonomically and annotated by prokka [48]. The profiles of multi-locus sequencing typing (MLST) were predicted by using mlst v2.10 (T. Seemann, https://github.com/tseemann/mlst) against the PubMLST database [49]. Searching SCC*mec* was performed using SCCmecFinder (https://cge.cbs.dtu.dk/services/SCCmecFinder/). The resistomes inferred from the draft genomes were analyzed by BLASTN [50] and ABRicate (T. Seemann, https://github.com/tseemann/abricate) searches against the ResFinder [51] and CARD [52]. The presence of virulence genes was inferred by BLASTN searches against the Virulence Factors DataBase (VFDB) [53].

### 4.5. Data Analysis

The association between *Staphylococcus* species (*S. aureus* and *S. pseudintermedius*) and AMR frequencies was assessed using Fisher’s exact test. The relationship between the origin of the sample (body fluids, ear exudates and other origins) and AMR were also evaluated using Fisher’s exact and multiple proportions Z-test with Bonferroni adjustment for multiple comparisons, respectively. The association between the resistance rates to the different antibiotics tested was determined by Cohen’s kappa coefficient (*κ*). The Cochran–Armitage trend test was used to determine the increase in different AMR over time. Statistical tests were performed using SPSS 25 software (IBM, New York, NY, USA), except the Cochran–Armitage trend test, which was performed with the WinPepi package [54]. Statistical significance was established at *P* < 0.05.

## 5. Conclusions

In conclusion, the results of this study confirm the value of MALDI-TOF technologies for species-level identification of SIG isolates of animal origin. Furthermore, we observed a high prevalence of MDR and resistance to fluoroquinolones, cephalosporins and macrolides, and detected epidemic MRSP genotypes such as ST71 among isolates from dogs. Finally, we found a variety of genes that have been related to antibiotic-resistant phenotypes in the genome of MRSP isolates. All in all, these results highlight the emerging threat posed by MRSP and the need for continuous surveillance of this bacterial group for antimicrobial resistance in the veterinary setting.

## Figures and Tables

**Table 1:** Comparison of results of the species-level identification of coagulase-positive *Staphylococcus* (CoPS) isolates (*n* = 139) yielded by multiplex species-specific PCR, VITEK and MALDI-TOF.

Species	PCR ^a^	VITEK	MALDI-TOF ^b^
*Staphylococcus aureus*	NA ^c^	32	19
*Staphylococcus pseudintermedius*	117	19	112
*Staphylococcus intermedius*	0	88	4
*Staphylococcus schleiferi*	3	0	3

^a^ Sasaki et al., 2010 (based on partial *nuc* gene to differentiate among *Staphylococcus intermedius* group (SIG) members) performed as multiplex PCR systems. This PCR was considered the gold standard technique for SIG identification in the present study. ^b^ One isolate was identified as *Staphylococcus* spp. ^c^ Not applicable, identification of *S. aureus* was performed by VITEK and MALDI-TOF. MALDI-TOF was considered the gold standard technique for identification of this species.

**Table 2:** Resistance frequency of 19 *S. aureus* and 117 *S. pseudintermedius* isolates recovered at the Complutense Veterinary Teaching Hospital (Spain) between 2006 and 2017.

Antibiotic Class	Antibiotic (Abbreviation)	Disk Content (µ)	Breakpoint	Number of Resistant (% Resistance)	
				*S. aureus* (n = 19)	*S. pseudintermedius* (n = 117)
*Fluoroquinolones*	*Ciprofloxacin (CIP) ^a^*	5	≤15	3(15.79)	23(19.65)
	*Ofloxacin (OFX) ^a^*	5	≤14	4(21.05)	22(18.80)
	*Norfloxacin (NOR) ^a^*	10	≤12	5(26.31)	22(18.80)
*Trimethoprim-sulfamethoxazole*	*Trimethoprim-* *Sulfamethoxazole (SXT) ^a^*	25	≤10	0	35(29.21)
*Lincosamide*	*Clindamycin (CLI) ^a^*	2	≤14	3(15.79)	50(42.73)
*Cephalosporins*	*Cefoxitin (FOX) ^a^*	30	≤24	4(21.05)	9(7.69)
*Tetracyclines*	*Tetracycline (TET)* *^a^*	30	≤14	2(10.53)	60(51.28)
	*Doxycycline (DOX) ^a^*	30	≤12	0	0
*Penicillins*	*Penicillin (PEN)* *^a^*	6	≤28	17(89.47)	104(88.89)
	*Oxacillin ^b^*	-	≥0.5/≥ 4 ^c^	4(21.05)	19(16.23)
*Aminoglycosides*	*Kanamycin (KAN) ^a^*	30	≤13	3(15.79)	58(49.57)
	*Amikacin (AMK) ^a^*	30	≤14	3(15.79)	1(0.85)
	*Gentamicin (GEN) ^a^*	10	≤12	3(15.79)	28(23.93)
*Macrolides*	*Erythromycin (ERY) ^a^*	15	≤13	5(26.31)	54(46.15)
	*Clarithromycin (CLR) ^a^*	15	≤13	5(26.31)	54(46.15)
*Chloramphenicol*	*Chloramphenicol (CHL) ^a^*	30	≤12	0	29(24.78)
*Teicoplanin*	*Teicoplanin (TEC)* *^a^*	30	≤10	0	0
*Linezolid*	*Linezolid (LZD)* *^a^*	30	≤20	0	0
*Rifampin*	*Rifampin (RIF)* *^a^*	RIF5	≤16	0	0
Vancomycin.	*Vancomycin ^b^*	−	≥32/≥16 ^d^	0	0

^a^ Antibiotic susceptibility was determined by the disk diffusion technique; zone diameters were expressed in mm. ^b^ MICs were determined by agar dilution and are expressed in µg/mL. ^c^ Breakpoint for Coagulase-negative *Staphylococcus* (0.5) and *S. aureus* (4). ^d^ Breakpoint for Coagulase-negative *Staphylococcus* (32) and *S. aureus* (16).

**Table 3:** Rates of antimicrobial resistance of 3 *S. schleiferi*, 19 *S. aureus* and 117 *S. pseudintermedius isolates* recovered at the Complutense Veterinary Teaching Hospital (Spain) during different periods (2007–2010, *n* = 44; 2011–2014, *n* = 44 and 2015–2016, *n* = 51).

Antibiotic (Abbreviation)	Disk Content (µg)	Number of Resistant (% Resistance)		
		2007–2010 (n = 44)	2011–2014 (n = 43)	2015–2016 (n = 51)
*Kanamycin (KAN)*	30	15 (34.09%)	20 (45.45%)	26 (50.98%)
*Amikacin (AMK)*	30	1 (2.27%)	2 (4.55%)	1 (1.96%)
*Gentamicin (GEN)*	10	6 (13.64%)	10 (22.73%)	15 (29.41%)
*Cefoxitin (FOX)*	30	3 (6.82%)	6 (13.64%)	4 (7.84%)
*Penicillin (PEN)*	6	38 (86.36%)	36 (81.82%)	47 (92.16%)
*Ciprofloxacin (CIP)*	5	4 (9.09%)	8 (18.18%)	15 (29.41%)
*Norfloxacin (NOR)*	10	5 (11.36%)	8 (18.18%)	15 (29.41%)
*Ofloxacin (OFX)*	5	4 (9.09%)	8 (18.18%)	15 (29.41%)
*Tetracycline (TET)*	30	21 (47.73%)	22 (50%)	19 (37.25%)
*Erythromycin (ERY)*	15	18 (40.91%)	17 (38.64%)	24 (47.06%)
*Clarithromycin (CLR)*	15	18 (40.91%)	17 (38.64%)	24 (47.06%)
*Teicoplanin (TEC)*	30	0	0	0
*Doxycycline (DOX)*	30	0	0	0
*Trimethoprim-* *Sulfamethoxazole (SXT)*	25	4 (9.09%)	16 (36.36%)	15 (29.41%)
*Linezolid (LZD)*	30	0	0	0
*Rifampin (RIF)*	5	15 (34.09%)	17 (38.64%)	21 (41.18%)
*Clindamycin (CLI)*	2	0	0	0
*Chloramphenicol (CHL)*	30	10 (22.73%)	8 (18.18%)	11 (21.57%)

## Supplementary Materials

The following are available online at https://www.mdpi.com/2079-6382/9/11/752/s1, Table S1: Phenotypes and molecular AMR profiles of 20 Methicillin-Resistant Coagulase Positive *Staphylococcus* recovered from animals in Spain.
